# Comparison of bactericidal and cytotoxic activities of trichogin analogs

**DOI:** 10.1016/j.dib.2015.12.006

**Published:** 2015-12-17

**Authors:** Regina Tavano, Giulia Malachin, Marta De Zotti, Cristina Peggion, Barbara Biondi, Fernando Formaggio, Emanuele Papini

**Affiliations:** a Department of Biomedical Sciences, University of Padova, Viale G. Colombo 3, 35121 Padova, Italy; b Institute of Biomolecular Chemistry, CNR, Padova Unit, Department of Chemistry, University of Padova, Via Marzolo 1, 35131 Padova, Italy

**Keywords:** Peptaibols, Trichogin, Antibaterial activity, Cytotoxicity

## Abstract

Peptaibiotics are a group of membrane active peptides of fungal origin. They typically contain α-aminoisobutyric acid (Aib; 1-letter code, U) and other non-coded residues (Toniolo and Brückner, 2009; Neumann et al., 2015; Benedett et al., 1982) [1], [2], [3] stabilizing their helical structure. Peptaibols are peptaibiotics carrying a 1, 2-aminoalcohol at the C-terminus. When a fatty acid chain (of 8–10 carbon atoms) is present at their N-terminus, they are called lipopeptaibols (Toniolo et al., 2001; Degenkolb et al., 2003) [4], [5]. We found (Tavano et al., 2015) [6] that the lipopeptaibol trichogin displays no antibacterial effects up to 64 µM, against both Gram^−^ and Gram^+^ bacteria, but kills tumor and healthy human cells via a mechanism requiring both the C-terminal primary alcohol group and the N-terminal n-octanoyl moiety, with EC50s around 4–5 µM. However, the substitution of single Gly residues with Lys strongly improves anti-Gram^+^ activity (Tavano et al., 2015; De Zotti, Biondi, Park et al., 2012; De Zotti, Biondi, Peggion et al., 2012) [6], [7], [8]. To further characterize the activity of trichogin analogs as antibiotics and cytotoxic agents, we here manipulated the peptide helix amphipathicity by means of two different substitutions: (i) Aib to Leu (De Zotti et al., 2012) [7] or (ii) multiple Gly to Lys changes (Tavano et al., 2015; De Zotti, Biondi, Park et al., 2012; De Zotti, Biondi, Peggion, Formaggio et al., 2012; De Zotti, Biondi, Peggion, De Poli et al., 2012) [6], [7], [8], [9]. The antibacterial activity against four commensal or opportunistic bacterial species and the cytotoxicity against a panel of 9 healthy and tumor-derived eukaryotic cell types (including erythrocytes) are reported as MIC and EC50 (MTS - [3-(4, 5-dimethylthiazol-2-yl)-5-(3-carboxymethoxyphenyl)-2-(4-sulfophenyl)]-2H-tetrazolium- reduction and LDH - lactate dehydrogenase - release assay).

**Specifications Table**

**Table:** 

Subject area	*Chemistry, Biology*
More specific subject area	*Peptaibols*
Type of data	*Table, text file, graph, figures*
How data was acquired	*HPLC, mass spectrometry, FACS, spectrofluorimetry*
Data format	*Analyzed*
Experimental factors	*Different kind of bacterial or eukaryotic cells were treated with different concentrations of peptides for* 24 h
Experimental features	*After incubation with peptides, cell viability* (*MTS assay*)*, type of death* (*Annexin*-*propidium iodide assay*) *or fluorescence associated to the cells* (*FACS analysis*) *were assessed*
Data source location	*University of Padova, Italy*
Data accessibility	*The data are supplied with this article*

**Value of the data**

• The need to find new antibacterial agents able to overcome antibiotic resistance and, at the same time, the expectation of having new effective antitumor drugs may benefit from the accurate quantification of the antimicrobial and cytotoxic efficacy and selectivity of peptaibols.
• We here report quantitative data from which it is possible to compare the antibiotic activity and the cytocidal efficacy of several analogs of trichogin GA IV.
• The data reported here may be useful since they provide a base for possible further design and refinement of either anti-microbial or anti-tumor activity of trichogin-derived peptides.

## 1 Data

### 1.1 Antibacterial activity of trichogin and its analogs

As previously observed [7], analogs L1 and L1,8 (Table 2; throughout text, the 1-letter code for amino acids was used) showed a moderately improved anti *Staphylococcus aureus* (*S. aureus*) activity (MIC after 24 from >64 μM to 32 μM), whilst L4, known to have a destabilized helix [7], showed no such increase. Introduction of one, two or three K residues in the sequence (Table 1) induced a strong MIC (24 h) decrease against the Gram^+^ bacteria tested (2–5 μM) and the Gram^−^ *Escherichia coli* (*E. coli*) (8–16 μM) (Table 2). Anti-Gram^−^ *Pseudomonas aeruginosa* (*P. aeruginosa*) activity was also improved in di- or tri-K containing analogs (MIC, 12–16 μM). To further improve the antibacterial activity of the K-containing analogs we introduced an additional U residue at position 6, to stabilize the helix (K5U6, K2K5K9U6). In addition, we replaced U at position 1 with L (L1K2, L1K9) as this substitution was shown to improve the activity of trichogin against *S. aureus* [7]. However, these modifications failed to increase the antibacterial activity (Table 2).

### 1.2 Cytotoxic effects of trichogin analogs with multiple G to K substitutions

**Fig. 1:**
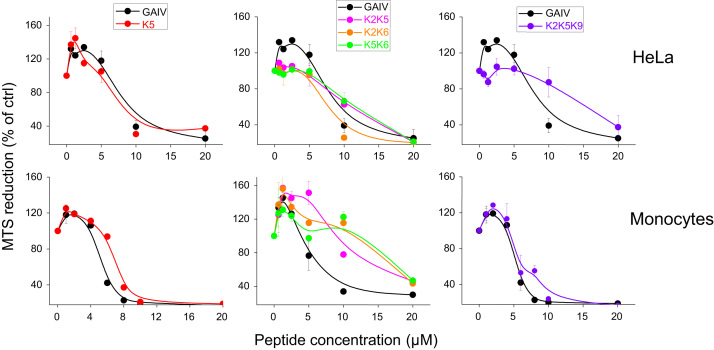
Cytotoxicity on human cells of trichogin and its analogs with G to K modifications. HeLa and HL60 cells were incubated for 24 h with the peptides at different concentrations and subjected to MTS assay. The values, expressed as percentage of control, are the mean±SD of three experiments run in duplicate. *S. epidermidis*: *Staphylococcus epidermidis*.

**Fig. 2:**
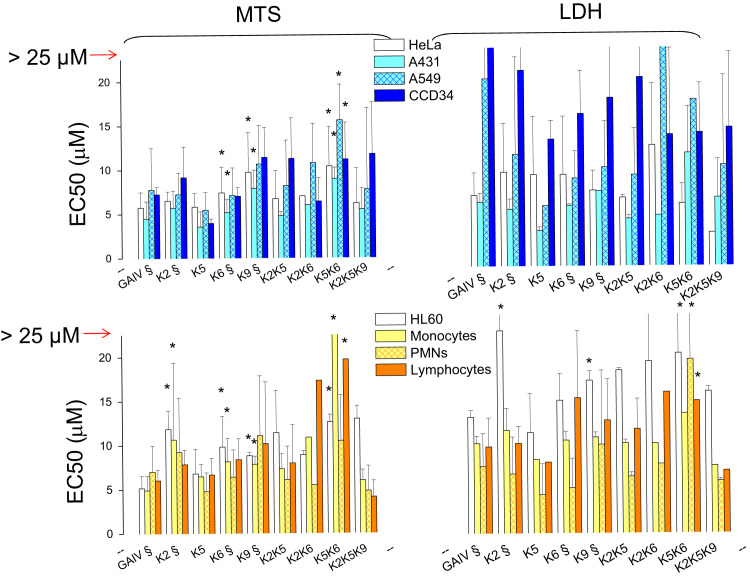
Cytotoxicity on human cells of trichogin and it analogs with G to K modifications. Cells treated with increasing doses of the peptides were subjected to MTS assay (A) or LDH assay (B). EC50 values of the cells treated with the peptides for 24 h, based on MTS and LDH assays, are the mean of four experiments run in duplicate±SD. *p<0.05 with respect to trichogin treated cells. § Indicates data from [6] for comparison.

To monitor the possible differential cytocidal effect of trichogin and its analogs on human cells (healthy and cancer cells, non-leukocytes and leukocytes), we incubated the peptides with (i) primary human leukocytes (lymphocytes, PMNs and monocytes), freshly isolated from blood of healthy donors (ii) the monocytes-like HL60 cell line from human myeloid leukemia, and (iii) three tumor-derived non-leukocyte cells lines: HeLa (human ovary sarcoma), A431 (human epidermoid carcinoma) and A549 (human lung carcinoma) and the *in vitro* stabilized line CCD34Lu (from human normal lung). MTS reduction rate (see Fig. 1 as an example) and LDH release were then measured after 24 h to obtain EC50 values (the dose of peptide leading to 50% effect). Among all K-containing peptides, only di-substituted K5K6 showed a significantly reduced cytocidal effect against all cell models (range: 50–60% inhibition) (Fig. 2).

### 1.3 Modulation of trichogin eukaryotic cytotoxicity by replacing U with L

**Fig. 3:**
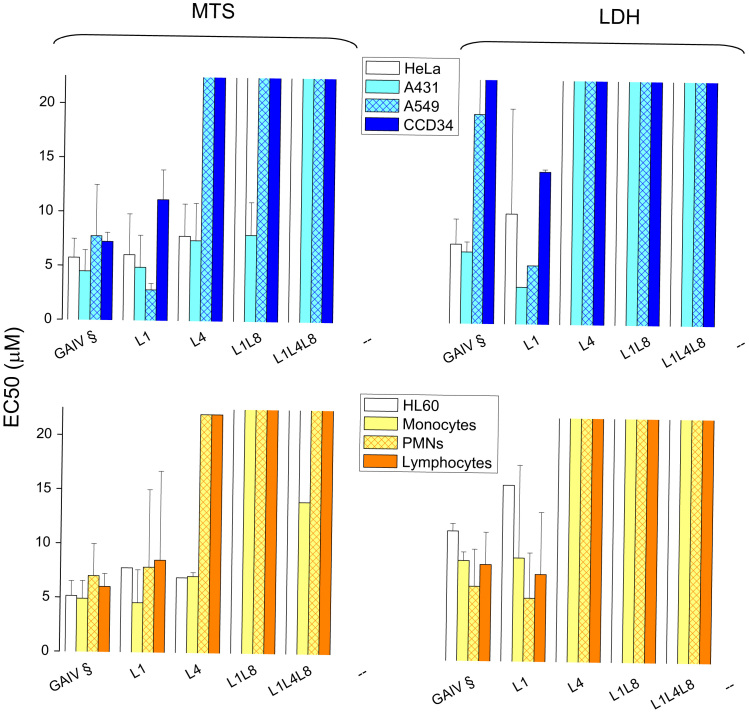
Cytotoxicity on human cells of trichogin and its U to L analogs. Cells treated with increasing doses of the peptides were subjected to MTS assay (A) or LDH assay (B). EC50 values of the cells treated with the peptides for 24 h, based on MTS and LDH assays, are the mean of four experiments run in duplicate±SD. § Indicates data from [6] for comparison.

**Fig. 4:**
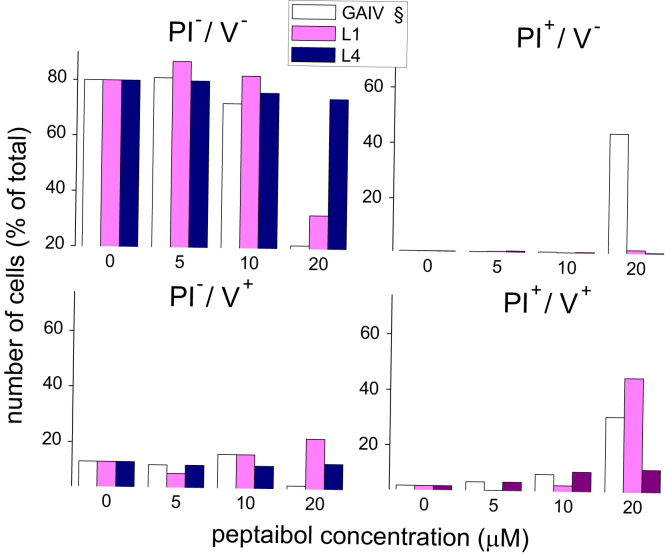
Induction of necrosis (PI^+^/V^−^), apoptosis (PI^−^/V^+^) or late apoptosis (PI^+^/V^+^) in HeLa cells treated with U to L analogs of trichogin. Cells treated as described in Fig. 3 were stained with Annexin-V-Fluorescein isothiocyanate (FITC) and Propidium iodide (PI), and subjected to FACS analysis. Bars are the percentage of viable (PI^−^/V^−^), necrotic (PI^+^/V^−^), apoptotic (PI^−^/V^+^) and late apoptotic (PI^+^/V^+^) cells from a representative experiment out of three. § Indicates data from [6] for comparison.

The cytotoxicity of native trichogin is weakly affected by replacing U^1^ with L (Fig. 3). The importance of a stable helical structure for the bioactivity of trichogin was further confirmed by the reduced cell toxicity (50–70% inhibition) of L4 [7] and L1L4L8 (no helicogenic U residues in the sequence) analogs. L4 was also found to be more toxic to HeLa cells than native trichogin. PI/Annexin V labeling experiments (Fig. 4) confirmed that L1 was as effective as trichogin in killing HeLa cells.

### 1.4 Modulation of K-substitued trichogins by L and U further substitution

**Fig. 5:**
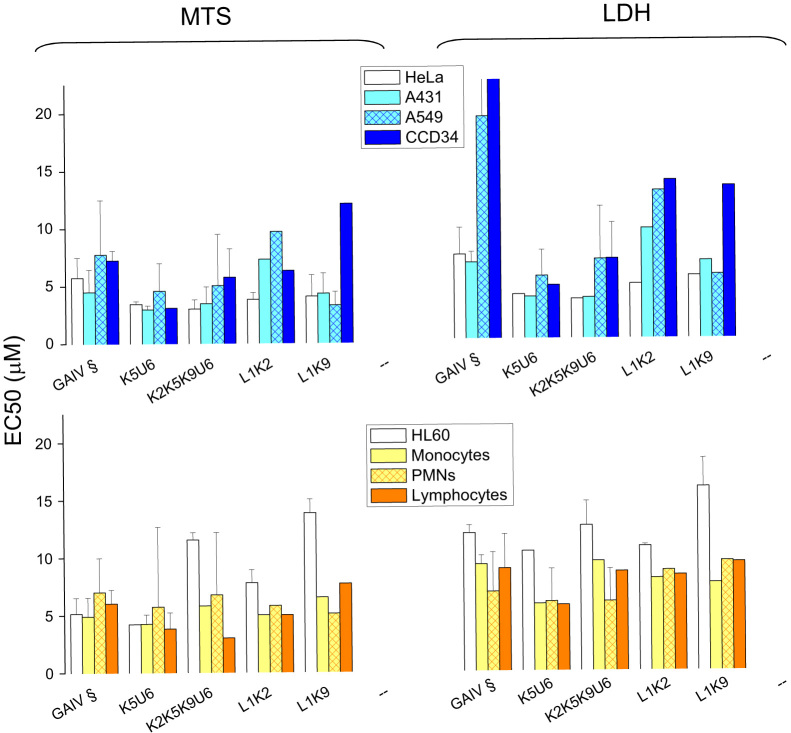
Cytotoxicity on human cells of K-substituted trichogins by L and U. Cells treated with increasing doses of the peptides were subjected to MTS assay (A) or LDH assay (B). EC50 values of the cells treated with the peptides for 24 h, based on MTS and LDH assays, are the mean of four experiments run in duplicate±SD. § Indicates data from [6] for comparison.

**Fig. 6:**
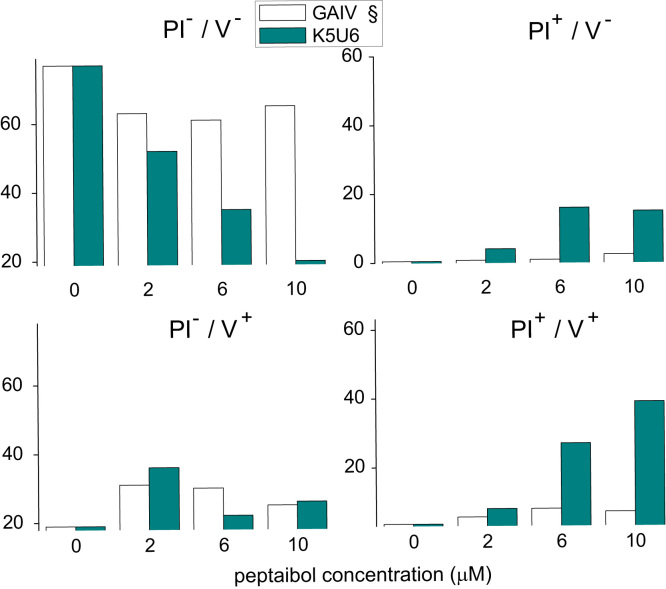
Induction of necrosis (PI^+^/V^−^), apoptosis (PI^−^/V^+^) or late apoptosis (PI^+^/V^+^) in HeLa cells treated with K5U6 analog of trichogin. Cells treated as described in Fig. 3 were stained with Annexin-V-FITC and Propidium iodide, and subjected to FACS analysis. Bars are the percentage of viable (PI^−^/V^−^), necrotic (PI^+^/V^−^), apoptotic (PI^−^/V^+^) and late apoptotic (PI^+^/V^+^) cells from a representative experiment out of three. § Indicates data from [6] for comparison.

Cytotoxicity assays were performed on the K-containing analogs L1K2, L1K9, K5U6, K2K5K9U6 (Fig. 5). In most cases the cytotoxic effect against all the tested cells was not significantly different from that of the corresponding analogs K2, K5, K9 and K2K5K9, with the only exceptions of K5U6 and K2K5K9U6, which showed an increased cytotoxicity. In agreement with MTS/LDH data, K5U6 was significantly more effective in killing HeLa cells as compared to either trichogin or K5. K5U6 induced predominantly a late apoptotic phenotype but also significant necrosis (Fig. 6).

### 1.5 Hemolytic activity of trichogin analogs

**Fig. 7:**
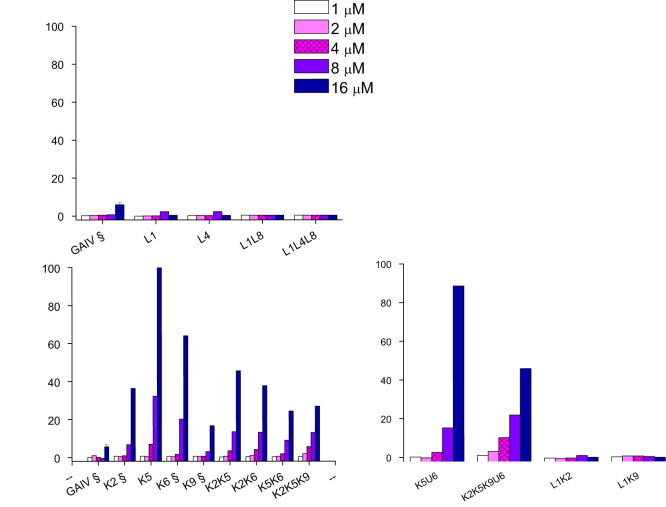
Hemolytic activity of trichogin and its analogs. Erythrocytes were treated for 24 h with different concentration of peptides, centrifuged and hemoglobin release was measured spectrophotometrically. Hemolysis is expressed as percentage relative to positive control (erythrocytes treated with distilled water). § Indicates data from [6] for comparison.

We found that trichogin is weakly hemolytic and that U-to-L substitutions did not change the hemolytic effect (Fig. 7). All K-containing analogs induced strong hemolysis (30–100%).

## 2 Experimental design, materials and methods

### 2.1 Peptide synthesis and characterization

The sequences of the trichogin analogs investigated are listed in Table 1. The newly synthesized peptides were prepared by solid-phase procedures, according to published protocols [9], [10]. After HPLC purification, their purity was >96%. The peptides were characterized by means of HPLC and mass spectrometry, as reported in Table 1.

### 2.2 Bacterial strains and antibacterial activity

*E. coli* ATCC 25922, the methicillin-resistant strain of *S. aureus*, ATCC BAA-44, *P. aeruginosa* ATCC 25668 and *S. epidermidis* ATCC 700565 was a kind gift of Prof. Elena Reddi (Dept. of Biology, University of Padova, Italy). Cultures were maintained in Luria Bertani (LB) agar. MICs (Minimal inhibitory concentrations) of the peptides were determined using the broth microdilution method. Two-fold serial dilutions of each peptide, from 1 to 64 μM, were prepared in LB and 50 μl per well were arranged in sterile 96-well plates (Falcon). Then, an aliquot of bacterial cell suspension was added to each well, at a final concentration of 5×10^5^ CFU/ml. After incubation for 24 h at 37 °C, the inhibition of bacterial growth was assessed and the MIC endpoint was defined as the lowest concentration of the antimicrobial peptide that completely inhibited bacterial growth.

### 2.3 Cell isolation and culture

HeLa, A431 and CCD34-Lu cells were maintained in DMEM medium (Gibco), A549 in F12 medium (Gibco) and HL60 in RPMI medium (Gibco), supplemented with 10% FCS (Euroclone) and antibiotics (penicillin and streptomycin, 100 U/ml, Invitrogen) at 37 °C in a humidified atmosphere containing 5% (v/v) CO_2_; cells were split every 2–3 days. Human monocytes, polymorphonuclear leukocytes (PMNs) and lymphocytes were purified from buffy coats of healthy donors, kindly provided by the Centro Immunotrasfusionale, Hospital of Padova. Briefly, for monocyte purification, peripheral blood mononuclear cells (PBMC) were isolated from buffy coats of healthy donors by density gradient centrifugation on Ficoll-Paque Plus (GE Health care), which density is optimized for the isolation of mononuclear cells. Separate monocyte and T-cell fractions were obtained from PBMCs by Percoll density gradient centrifugation (GE Health care); residual lymphocytes were removed by incubation in 2% fetal calf serum (FCS) RPMI at 37 °C and subsequently washed to eliminate non adherent cells. Unless otherwise specified, cells were kept at 37 °C in a humidified atmosphere containing 5% (v/v) CO_2_ in RPMI-1640 supplemented with 10% FCS. For PMNs purification, the pellet of cells obtained after the centrifugation on Ficoll gradient was subjected to dextran erythrocytes precipitation; residual erythrocytes were removed by hypotonic lysis in 155 mM NH_4_Cl, 10 mM KHCO_3_, and 100 mM Na_2_EDTA at pH 7.4 and cells were cultured in RPMI medium, supplemented with 10% FCS. For lymphocytes preparation, buffy coats were incubated with 50 μl/ml of Rosette Sep® Human T Cell Enrichment Cocktail (StemCell Technologies). Blood was then centrifuged over a Ficoll gradient and cells were cultured in RPMI medium, supplemented with 10% FCS.

### 2.4 MTS assay

Twenty-four hours before the experiment, A431, HeLa, A549 and CC34-Lu cells were detached by means of trypsin treatment (Gibco), counted and seeded onto a 96 wells/plate (8×10^3^ cells/well, Falcon). After purification, monocytes were seeded onto a 96 wells/plate (2×10^6^/well) and left to adhere to the plastic; then cells were immediately treated with stimuli. PMNs and lymphocytes (2×10^6^ cells/well) and HL60 cells (40×10^3^) were seeded onto cell plates and subsequently treated. Different peptides were diluted in culture medium (from 20 μM to 1 μM) and added to the cells. As a positive control cells were treated with medium without stimuli. Twenty-four hours after treatment, the cell supernatant was collected for subsequent assay (LDH assay) and cells were tested using a CellTiter 96® AQueous One Solution Reagent (Promega). Plates were read with an ELISA reader (Amersham Biosciences) at 492 nm. Data were expressed as % of absorbance value compared to untreated cells (assumed as 100% viable). Alternatively, the EC50 (the concentration of drug where 50% of cells died) was calculated for each peptide and each kind of tested cell.

### 2.5 LDH assay

50 μl of the cell supernatant was tested with a CytoTox 96® Non-Radioactive Cytotoxicity Assay kit (Promega). EC50 (the concentration of drug that induced the lysis of 50% of the total cell number) was calculated for each peptide and each kind of tested cell.

### 2.6 Propidium iodide and Annexin V assay

HeLa cells were seeded on 24-wells plastic trays (Falcon) the day before the experiment, while HL60 were used directly. Cells were treated for 18 h with different trichogin analogs (up to 20 μM); then they were washed with PBS, detached with trypsin (in the case of HeLa cells), centrifuged at 200*g* for 5 min, suspended in 50 μl of incubation buffer and incubated for 15 min at 37 °C with 2 μl of Annexin-V-Fluos (Roche). Cells were finally diluted in 250 μl of the incubation buffer and 15 μl of Propidium Iodide (Sigma) were added to each sample just before the acquisition with FACSCanto® (Beckton Dickinson). Percentage of healthy (Annexin V^−^/PI^−^), apoptotic (Annexin V^+^/PI^−^), oncotic (Annexin V^−^/PI^+^) and late apoptotic/abortive oncotic (Annexin V^+^/PI^+^) cells of the total (10^6^ cells) was calculated using FACSDiva Software.

## Figures and Tables

**Table 1:** Trichogin and its analogs used in this work.

*Sequence*	*Name*	*Ref.*	[*M*+*H]*^+^*_calcd_*	[*M*+*H*]^+^*_found_*	*R_t_* (min)⁎
Oc-UGLUGGLUGILol	**GA IV**	[1]	–	–	–
Oc-LGLUGGLUGILol	**L1**	[4]	–	–	–
Oc-UGLLGGLUGILol	**L4**	[4]	–	–	–
Oc-LGLUGGLLGILol	**L1L8**	[1–4]	–	–	–
Oc-LGLLGGLLGILol	**L1L4L8**	This work	1150.81	1150.80	19.23^a^
Oc-UGLUK(HCl)GLUGILol	**K5**	[1]	–	–	–
Oc-UK(HCl)LUK(HCl)GLUGILol	**K2K5**	This work	1208.86	1208.91	12.93^b^
Oc-UK(HCl)LUGK(HCl)LUGILol	**K2K6**	This work	1208.86	1208.85	24.34^c^
Oc-UGLUK(HCl)K(HCl)LUGILol	**K5K6**	[1]	–	–	–
Oc-UK(HCl)LUK(HCl)GLUK(HCl)ILol	**K2K5K9**	This work	1279.94	1280.02	11.36^b^
Oc-UGLUK(HCl)ULUGILol	**K5U6**	This work	1165.82	1165.82	20.25^d^
Oc-UK(HCl)LUK(HCl)ULUK(HCl)ILol	**K2K5K9U6**	[1]	–	–	–
Oc-LK(HCl)LUGGLUGILol	**L1K2**	This work	1165.82	1165.79	22.19^e^
Oc-LGLUGGLUK(HCl)ILol	**L1K9**	This work	1165.82	1165.88	13.24^f^

Oc, octanoyl; U, Aib; Lol, leucinol.

⁎ HPLC retention time. Elution conditions: column, if not otherwise specified, Phenomenex Jupiter 300 Å, 5µ, C_18_; flow, 1 ml/min; eluent A: 9:1 H_2_O/CH_3_CN+0.05% TFA; eluent B: 9:1 CH_3_CN/H_2_O+0.05% TFA.

a Gradients used: 70–100%B in 15 min (column: Vydac 300 Å, 5µ, C4).

b Gradients used: 50–80%B in 20 min.

c Gradients used: 10–90%B in 30 min.

d Gradients used: 60–100%B in 22 min.

e Gradients used: 30–100%B in 30 min.

f Gradients used: 60–80%B in 20 min.

**Table 2:** MICs (μM±SE; *N*≥4; peptides dose range: 0–64 µM) after 24 h against two Gram^+^ and two Gram^−^ bacterial strains (§ indicates data from [6] for comparison).

Compound	*S*. *aureus*	*S*. *epidermis*	*P*. *aeruginosa*	*E*. *coli*
**GA IV (§)**	>64	>64	>64	>64
**L1**	32±0	>64	>64	>64
**L4**	>64	>64	>64	>64
**L1L8**	32±0	>64	>64	>64
**K2 (§)**	3±1.4	4.3±2	>64	24±17.1
**K5**	1±0	2±0	>64	8±2
**K6 (§)**	3±1.7	4±0	>64	16±0
**K9 (§)**	5.3±2.1	4.7±1.6	57±16	32±17.5
**K2K5**	3.3±1.3	5±2.4	16±5	13.7±3.9
**K2K6**	2.8±1.1	3.2±2.6	17.6±8.8	12.6±4.3
**K5K6**	4±0	4±1	16±0	16±0
**K2K5K9**	4.3±1.8	4±0.9	13.3±4.1	14.9±8.6
**K5U6**	3.8±2.4	2.4±0.9	>64	>64
**K2K5K9U6**	4±0	8±0	8±0	8±0
**L1K2**	4.6±1.5	4.7±1.6	>64	22.9±8.6
**L1K9**	4.7±1.6	4.6±1.5	>64	41.1±15.6
